# Specialist breast cancer nurses’ views on implementing a fear of cancer recurrence intervention in practice: a mixed methods study

**DOI:** 10.1007/s00520-019-04762-9

**Published:** 2019-04-17

**Authors:** Susanne Cruickshank, Emma Steel, Deborah Fenlon, Jo Armes, Elspeth Banks, Gerald Humphris

**Affiliations:** 1 Faculty of Health Sciences and Sport, University of Stirling, Stirling, FK9 4LA Scotland; 2 College of Health and Human Sciences, Swansea University, Swansea, SA2 8PP UK; 3 School of Health Sciences, University of Surrey, Guildford, GU2 7XH UK; 4 National Cancer Research Institute, Angel Building 407 St John Street, London, EC1V 4AD UK; 5 School of Medicine, University of St Andrews, St Andrews, KY16 9TF UK

**Keywords:** Breast cancer, Fear of cancer recurrence, Normalisation process theory, Mixed methods, Mini-AFTERc intervention, Nurse, Nurse specialist

## Abstract

**Introduction:**

Fear of cancer recurrence (FCR) in people with breast cancer affects treatment recovery, quality of life, service utilisation and relationships. Our aim was to investigate how specialist breast cancer nurses (SBCN) respond to their patients’ fears of cancer recurrence and analyse SBCN’s views about embedding a new psychological intervention, the Mini-AFTERc, into their consultations.

**Method:**

A mixed methods sequential design was used, informed by normalisation process theory. Phase 1: UK SBCNs were emailed a web-based survey to investigate how breast cancer survivors’ FCR is currently identified and managed, and their willingness to utilise the Mini-AFTERc. Phase 2: a purposive sample of respondents (*n* = 20) were interviewed to augment phase 1 responses, and explore views on the importance of addressing FCR, interest in the Mini-AFTERc intervention, its content, skills required and challenges to delivering the intervention.

**Results:**

Ninety nurses responded to the survey. When SBCN’s were asked to identify the proportion of patients experiencing FCR in their caseload, there was no consensus on the size of the problem or unmet need. They estimated that 20–100% people experience moderate FCR and 10–70% severe FCR. The interviews identified that clinical conversations are focused primarily on giving information about signs and symptoms of recurrence rather than addressing the psychological aspects of fear.

**Conclusion:**

Findings indicate wide variability in how FCR was identified, assessed and supported by a sample of UK SBCNs. The introduction of a structured intervention into practice was viewed favourably and has implications for nursing and health professional ways of working in all cancer services.

**Electronic supplementary material:**

The online version of this article (10.1007/s00520-019-04762-9) contains supplementary material, which is available to authorized users.

## Introduction

Survival outcomes for people with breast cancer have improved globally over the past 20 years [1]. Despite this, people remain at risk of metastatic recurrence (local and distant) up to, and beyond, 20 years after diagnosis [2]. The risk is strongly correlated with the original tumour, nodal status and tumour grade. The uncertainty of this risk enables fears of cancer recurrence to become established among breast cancer survivors [3–6].

Fear of cancer recurrence (FCR) is defined as “fear, worry, or concern about cancer returning or progressing” [3]. It is a natural response to a cancer diagnosis and experienced on a continuum from none to severe [6]. It is one of the most frequent unmet needs reported in the immediate post-treatment phase [7]. When severe, it can lead to distress, difficulty coping and poorer physical health [8], leading many to seek help from clinicians such as doctors and specialist breast cancer nurses [9–12]. FCR can be exacerbated once treatment is complete due to ongoing side effects, and can increase healthcare usage as women seek reassurance in distinguishing between recurrence and treatment-related bodily change or symptoms [13, 14].

A recent systematic review [15] highlighted the need for interventions to assist people with cancer to manage their increased levels of fear and a number of intensive psychological interventions have been developed, e.g. AFTER [16], CONQUER [17], SWORD [18]. Whilst there is some evidence of their effectiveness in people with severe FCR, they are resource intensive and therefore availability may be limited. Given the large numbers diagnosed with breast cancer, and approximately 30–70% experiencing moderate to severe FCR, nurses, clinical psychologists and psychiatrists need to find ways to utilise their different skills to address this increasing concern [15]. Humphris developed a shorter version of the AFTER intervention—the Mini-AFTERc. It consists of a single 30-min structured phone call designed to be made by a specialist breast care nurse (SBCN) instead of the six session AFTER intervention. Whilst not eradicating FCR completely, it aims to reduce it to a level that does not significantly interfere with daily life [19]. The Mini-AFTERc consists of a set of recommended questions to assess relevant issues related to FCR triggers, intensity, frequency and consequences. Once this brief assessment is completed, the SBCN selects one or more to (a) ascertain its significance and impact on the patients’ everyday life, (b) the nature of the symptom(s), (c) triggers of FCR following treatment and (d) identifying potential confidantes in the family or friends, and/or if there are difficulties discussing fears. Our previous experience [19] found that typically a single aspect is highlighted for attention during the phone call; however, additional issues can also be raised. The Mini-AFTERc manual provides written instruction, with diagrammatic figures to aid comprehension and includes a number of examples. We are currently conducting a pilot Mini-AFTERc at four cancer centres (HIPS/17/57).

In many countries, SBCNs, or clinicians in similar roles, are key providers of emotional and psychological support in clinical practice and may witness daily expressions of FCR by women with breast cancer [20–22]. Nevertheless, it is unclear how SBCNs identify, support and address concerns of people with FCR in their daily work and if they view this as part of their role. Moreover, prior to assessing the efficacy of the Mini-AFTERc, we decided it would be prudent to ascertain whether SBCNs believe and have the capacity to deliver it as part of their clinical practice [23].

The aim of this study was to determine the SBCN views on implementing the Mini-AFTERc intervention into their practice. The objectives were to:

1. Capture current approaches used by SBCNs to identify and manage FCR
2. Identify challenges and barriers experienced by SBCNs in assessing and managing FCR
3. Assess SBCNs willingness to implement the Mini-AFTERc intervention and understand what would enable successful implementation

## Methods

### Study design

We conducted a sequential explanatory mixed methods study. Detailed information on the study design and protocol has been published previously [24]. This design has two phases: (1) Quantitative survey and (2) qualitative interviews. The qualitative data were collected subsequent to the quantitative data to elaborate on the survey responses in the first phase [25]. The overall design was informed by normalisation process theory (NPT). NPT seeks to identify the component parts for understanding and evaluating the implementation process that enable an intervention (e.g. Mini-AFTERc) not only to be operationalised and normalised into everyday work (embedded), but also sustained in practice (integration) [26]. It comprises four key constructs: (a) coherence, the sense-making work people do when faced with a new set of practices; (b) cognitive participation, the relational work people do to build and sustain a community of practice around a new technology or complex intervention; (c) collective action is the operational work people do to enact a set of practices; and (d) reflexive monitoring, the appraisal activity people do to assess and understand how new set of practices affect themselves and others.

In phase 1, a web-based survey using the Bristol online survey tool [27] was emailed to health professionals, between November 2017 and February 2018, who were registered with Breast Cancer Care’s Nursing UK Network [28].

In phase 2, semi-structured telephone interviews with SBCNs who indicated a willingness to be interviewed on the survey were completed. Prior to interview, an information pack about the Mini-AFTERc was distributed to participants, including an online presentation and extracts from the manual. The interview explored in-depth survey responses and, in particular, the importance of addressing FCR in clinical consultations, interest in the Mini-AFTERc intervention, skills required and challenges to intervention delivery.

The study was approved by the University of Stirling’s Research Ethics committee (SREC 15/16-paper no.65). To maintain confidentiality and anonymity of survey participants, Breast Cancer Care UK [28] distributed the survey via email.

### Participants

Clinicians registered with Breast Cancer Care’s Nursing Network (*n* = 905) were invited by email to complete the survey, of which 65% are estimated to be SBCNs (*n* = 588). A prerequisite of joining the network is that a clinician must spend 50% of their time working with people with breast cancer. There is no UK national SBCN register but, based on 50,000 new breast cancer cases in the UK, and the recommendation of one SBCN for every 100 people diagnosed, this network appears to represent most of them.

Data saturation (i.e. no new themes/issues raised) was achieved after 16 interviews. However, we continued to 20 (planned sample size) to ensure maximum variation on the sample. We developed a purposive sampling matrix (Online Resource 1) that ensured maximum variation in age, clinical focus, likelihood, or not, of discussing FCR with people and how comfortable, or not, they were discussing FCR.

### Phase 1: web-based survey

SBCNs were asked to complete a 35-item survey. The survey was divided into three sections. Questions 1–14 collected demographic data, role clarification including proportion of role spent doing clinical, research, administrative duties, education and training, and proportion of clinical work providing physical, social, spiritual and psychological support. Using the FCR definition [3], questions 15–24 focused on nurses’ perception of the level of FCR among patients seen in their daily practice, how they assessed and responded to FCR and how comfortable they were exploring these concerns. Questions 25–35 asked about support they received from their team and wider organisation, their interest in learning to deliver the Mini-AFTERc and their preferred support and training needs.

### Phase 2: qualitative interviews

The topic guide for the semi-structured interviews was developed using the normalisation process theory (NPT) framework [26] (Online Resource 2). Building upon survey responses, questions focused on the study objectives, whilst aligning these with the four NPT main components; coherence (e.g. whose responsibility is it to discuss FCR? Is there a shared sense of purpose to address FCR among people with breast cancer?), cognitive participation (e.g. would the SBCNs be willing to invest time and energy to attain competence with the intervention, investing time and energy), collective action (e.g. how would Mini-AFTERc affect workload and/or change the relationship between the patient and the SBCN?) and reflexive monitoring (e.g. questions about the perceptions of benefit to patient or staff, necessary requirements to make the intervention workable in practice). The interviews were conducted by telephone (ES), audio-recorded and transcribed verbatim.

### Data analysis

Survey data were analysed descriptively. Frequencies are presented as percentages.

All qualitative interviews were managed using NVivo 10 [29]. Framework analysis guided data analysis following a five step process [30]: (1) reading of the transcripts allowed for familiarisation with the data; (2) a thematic frame was developed based on NPT components and issues arising from the data; (3) the whole data corpus was then coded in relation to these specific themes; (4) charting of the data was conducted; and (5) mapping and interpretation of data within and across the themes allowed for interpretation and analysis. We utilised the NVivo comparison query [29] to permit ES to double code 25% of the transcripts to increase rigour and trustworthiness. ES and SC reviewed quotes and consensus was achieved on any statements not easily fitting the thematic framework, and shared them with the research team.

The weighting of the quantitative and qualitative data in a sequential explanatory design can vary depending on the study aims [26]. In this study, both were given equal weighting due to the additional information provided to the interviewees about the Mini-AFTERc intervention.

### Patient and public involvement

The GRIPP2 short form guided our reporting of public involvement (PPI) [31]. Our patient representative and co-author (EB) and the UK-based patient focused charity, Breast Cancer Care, shared their experience of supporting people with breast cancer using their helpline. Concerns about FCR emerge frequently as a topic women wish to discuss on their helpline call. This insight helped the researchers develop and conduct this study. They participated in all research meetings, online communications, and contributed to discussions that created a consensus among the research team. All iterations of documentation were reviewed and edited by all authors and collaborators.

## Results

### Phase 1

The invitation email was opened by 314 registrants (35%), of these 144 clicked on the survey (45%) and 90 completed the survey (29%). The demographic characteristics of the participants in both phases are presented in Table 1. Most were > 40 years (90%) and had been qualified > 11 years (91%, *n* = 82).

**Table 1:** Participant demographics—phases 1 and 2

Variable		Phase 1: % Survey respondents (*n*) *n* = 90	Phase 2: % Interviewees (*n*) *n* = 20
Age (years)	Under 30	1 (1)	0 (0)
	30–39	8 (9)	1 (5)
	40–49	33 (37)	7 (35)
	50–59	40 (44)	12 (60)
	60 and over	8 (9)	0 (0)
Number of years qualified as a nurse	Less than 2	0 (0)	0 (0)
	2–5	0 (0)	0 (0)
	6–10	8 (9)	0 (0)
	11–20	15 (17)	2 (10)
	21–30	36 (40)	11 (55)
	Over 30	31 (34)	7 (35)
Number of years working with people affected by breast cancer	Less than 2 2–5	2 (2) 8(9)	0 (0) 2 (10)
	6–10	16 (18)	3 (15)
	11–20	41 (46)	11 (55)
	21–30	22 (24)	4 (20)
	Over 30	1 (1)	0 (0)
Job banding	5	2 (2)	1 (5)
	6	22 (24)	6 (30)
	7	50 (56)	7 (35)
	8	14 (16)	6 (30)
	9	2 (2)	0 (0)
Clinical area	Surgery	41 (46)	8 (40)
	Oncology	24 (27)	8 (40)
	Surgery and Oncology	10 (11)	3 (15)
	Oncology and medical	2 (2)	0 (0)
	Surgery, oncology and medical	2 (2)	0 (0)
	Primary care	1 (1)	1 (5)
	Other	10 (11)	0 (0)

**Fig. 1:**
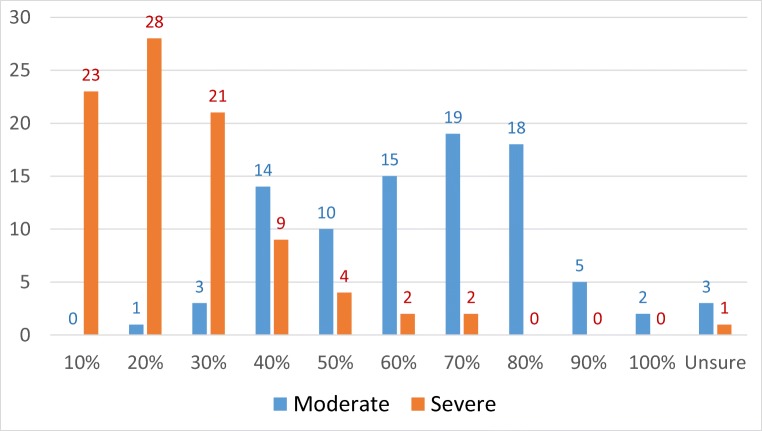
SBCN estimates of people with severe/moderate FCR in their caseload

When SBCNs were asked to identify the proportion of patients experiencing FCR in their caseload, there was no consensus as to the size of the problem or unmet need (see Fig. 1). Over half (*n* = 52) estimated 60–80% experienced moderate FCR (range 20–100%). Estimates for severe FCR were more conservative with most (*n* = 72) reporting they believed 10–30% (range 10–70%) experienced it. Only 14% reported initiating discussions about FCR with all their patients, whilst 45% said they would only discuss FCR if the patient raised it. Less than a fifth said they did not feel comfortable discussing FCR with patients. Most did not use a formal tool to assess FCR (78%) and reported in a free text box that they generally assessed FCR through discussion, open questions or informally at post-treatment clinics. Of 22 who reported using a formal tool, the most common measures included Macmillan Concerns Checklist (*n* = 13), FCR-specific scale (*n* = 2), departmental tool (*n* = 2) and other (*n* = 6). Main barriers to their use were time and patient compliance.

In the survey responses (Table 2), nurses reported they responded daily to patients’ concerns through various means. However, the formal psychological training and support they received to fulfil their role, and the perceived benefits gained, varied. Almost 50% reported that, with the exception of supportive team discussions, the benefits of attending clinical supervision or formalised team discussions were variable. Clinical supervision was important to some, both alone and with their team, but not everyone reported it helped them perform their role better. Of 19% who received training about FCR, most (*n* = 16) found it beneficial. When asked about training preferences to deliver the Mini-AFTERc, most (67%) were in favour of receiving training. Of these, 84% preferred face to face delivery, 75%, online delivery and 52% shadowing an experienced practitioner.

**Table 2:** Survey responses from the SBCNs

Different types of work undertaken by SBCNs on a daily basis							
	% of 90 (*n*)						
Anxiety management	13 (73)						
Communicating news	13 (74)						
Counselling	8 (46)						
Crisis interventions	7 (40)						
Supporting clinical choices	15 (84)						
Dealing with distress	15 (64)						
Information giving	15 (89)						
Preparing for treatment	14 (79)						
Nurses who report using assessment tools in their daily work							
	Yes	No	Maybe				
% (*n*) nurses using a FCR assessment tool	22 (20)	78 (70)	n/a				
% (*n*) nurses assessment tools (general)	28 (25)	72 (65)	n/a				
% nurses who find assessment tools helpful	70 (63)	30 (27)	n/a				
% (*n*) nurses willing to attend a Mini-AFTERc training course	74 (67	0	26 (23)				
Nurses who report receiving training/support to perform their role							
	SD	CS-1	CS-T	ITD	FTD	FCR-T	OPT
% nurses receiving training/support to perform their role	72 (64)	18 (24)	24 (31)	51 (68)	7 (9)	n/a	n/a
% nurses receiving specific FCR training						21 (19)	
Nurses perceived benefits of receiving different support/training to their work							
	< 50%	*n*	> 50%	*n*	n/a %	n/a *n*	
Support received from team SD	28	25	63	58			
One to one/clinical supervision CS-1	20	18	29	24	51	46	
Team clinical supervision (CS-T)	20	18	35	31	46	41	
Informal team discussion (ITD)	25	23	69	62	7	6	
Formalised team discussion (FTD)	17	17	31	31			
Specific FCR training (FCR-T)	16	3	73	16			
Other focused psychological training (OPT)	72	16	16	3			

*SD*, general support from team; *CS-1*, clinical supervision/one to one; *CS-T*, clinical supervision team; *ITD*, informal team discussion; *FTD*, formal team discussion; *FCR-T*, FCR training; *OPT*, other psychological training

### Phase 2

The qualitative interviews offered an opportunity to explain survey responses and additional insights into implementation of Mini-AFTERc in practice. The thematic coding frame was informed by NPT (Table 3), and illustrative quotes are provided below, with further quotes as Online Resource 3.

**Table 3:** Thematic coding framework of interview data

NPT Component – Main theme	Sub-themes	Coding
**Coherence** Is the sense making work that people do individually and collectively when they are faced with a problem of operationalising some set of practices	Identifying FCR – how it is raised	Formal assessment
		Not always addressed
		Probing for silent concerns
	Timing of FCR discussion	End of treatment – 6 months after
		On-going
		variable
	Managing FCR (strategies)	Discussing signs and symptoms
		Signposting
		Open access follow-up
	Confidence	Confident
		Managing uncertainties
		Difficult to raise
**Cognitive participation** Is the relational work the people do to build and sustain a community of practice around a complex intervention or technology	Training format	Face to face
		online
	Training aspects	Action plan
		Advanced communication
	Willingness to invest time	Adding to skillset
		Whole BCN team
**Collective action** Is the operational work that people do enact a set of practices, whether these represent a new complex intervention or technology	Changing practice	Enhanced practice
		more awareness of FCR
		Fits well
	Perceived difference between Mini-AFTER and current practice	More structured and specific
		Triaging tool
		Helps alleviate fear
**Reflexive monitoring** Is the appraisal work that people do to assess and understand the ways that a new set of practices affect them and others around them	Workable in practice	Timing of discussion
		Timing involved
		Fitting with other tools
	Who would Mini-AFTERc benefit	Patients
		SBCNS
	Patient sustainability	Offer to all patients
		Which patients would be suitable

### Coherence

Coherence related to how FCR is raised in practice, timing of discussion, strategies and, confidence. Some nurses stated they only discussed the possibility of recurrence if the patient raised it as a concern, for fear of introducing it into the patients’ consciousness.

“It’s all about reassuring. It’s all about everything’s going to be absolutely fine and to bring in the fact that, oh, actually, well, you...it might come back, you know, goes against a lot of what the message that I’d say we’re trying to get across is how I interpret it”. SBCN12

Those nurses, however, who initiated FCR discussion believed they were validating a pre-existing concern that most, if not all, patients already experienced.

As identified in the survey, most disclosed that they felt comfortable discussing FCR with patients. However, discussion on how SBCNs manage FCR suggested that many may simply talk about being aware of signs and symptoms of recurrence, without addressing fear as a psychological entity in its own right. Indeed, some recognised a need but were unclear about how best to act.

“I think my concern is not knowing what I could do about it. I mean, I can refer people for therapy, for counselling, but I don’t know that that’s necessarily the thing that they need. They’re not necessarily depressed or have an anxiety state, they need to know how to deal with that one particular problem”. SBCN19

Strategies for assessing people’s unmet needs were limited. When assessment tools were used, most spoke reported using the “Macmillan concerns assessment tool” despite it not including specific questions about FCR.

“If there was an HNA [holistic needs assessment] for every patient who was being discharged to open access [follow-up] then you could perhaps pick up more cases of FCR through that way. Unless somebody brings it up at a clinical appointment, we don’t tend to explore it. Sometimes they do and you can have a small discussion but the time we have is limited at the moment”. SBCN 1

### Cognitive participation

Cognitive participation explored training, format, aspects and time investment. Whilst challenges of implementing a new intervention were discussed, most nurses welcomed additional training and saw broader benefit not just to the patient but also their own working practice:

“It’s harder now than it used to be back in the day, but I think if it’s something that is going to be of value and you feel it is going to be of value professionally. I think you pick and choose now which you feel is going to be beneficial to you in your role, and your patient experience and expectation”. SBCN11

However, the importance of the intervention fitting within their current working practice was stressed;

“We wouldn’t have the time to do a separate session, so we would have to integrate it into something we’re already doing when we’re already seeing them”. SBCN1

There was no consensus about when to introduce it, to whom and where;

“I don’t think there’s any particular group that are more fearful than others. So in an ideal world, it would be great if everybody was offered this opportunity, but that would be absolutely impossible because of the number of breast patients that we see. So whether it could be something that the BCNs talk about and say, you know, if at any time in the future you feel as though you’re feeling particularly concerned, then we can have a more in-depth conversation”. SBCN2

### Collective action

This theme explored changing practice, differences between the Mini-AFTERc and current practice and impact on patients. Nurses spoke about enhancing practice and raising awareness of FCR:

“I’m not a hundred per cent sure if it would change practice greatly. I think it would just make us more aware of what we need to address” SBCN 14

Others could envisage quite clearly mutual benefits from the structured approach, which they saw as acting as a triage tool,

“It’s a much more structured approach obviously and it sounds like it has the potential to be able to differentiate between those patients we think we can help and those that really need a far more structured intervention or psychologist or therapist approach” SBCN13

### Reflexive monitoring

This theme explored enablers to successful implementation of the Mini-AFTERc: timing, using it appropriately and adding to SBCNs’ skills:

“I think anything that benefits the patients, and makes the journey easier and also gives us more skills in dealing with recurrence and fear of recurrence has to be beneficial to both the patients, the nurses, and to the service”. SBCN16

Not all nurses felt comfortable and a number mentioned concerns about the intervention being delivered by telephone rather than face to face:

“I would like to use face-to-face, I prefer face-to-face interventions and I think there is lots of unspoken and body language and just people’s unspoken behaviours that tell a story, so I prefer that but I think it would be useful to have as a phone intervention as well”. SBCN3

Combining the data, Table 4 provides a summary of some of challenges and solutions that would need to be considered to optimise a future trial to test efficacy.

**Table 4:** Development and optimisation of Mini-AFTERc intervention/pilot trial using NPT (Adapted from Murray et al. 2010)

NPT components	Questions using a NPT approach	NPT analysis to improve trial design
Coherence	What is the relationship between knowing about FoR is a concern and identifying how a new intervention aligns with everyday practice?	The intervention, described in more detail for the interview participants, was easily understood and distinguishable from other interventionsthey delivered.
	What is the worth attributed to introducing a FoR intervention?	Fear of recurrence was a term very familiar to the SBCN and recognised by many as an area of concern among patients they meet.
	Is the intervention easily described?	Perception of the proportion patients with moderate to severe FCR may be over or under-represented. This indicated a gap in accuracy in current assessment approaches used and therefore estimation of perceived benefit.
	Is there a shared sense of purpose?	
	Who would the intervention benefit?	
	Are benefits likely to be valued by women with breast cancer?	
Cognitive participation	Are the target groups, people affected with breast cancer, and SBCNs likely to think it is a good idea?	For SBCN, the trial would provide an opportunity to gain new skills through protected training and positively viewed.
	What kind of skills do SBCNs have now when dealing with FoR concerns?	It is expected a structured intervention could improve the confidence of SBCNs
	Are SBCN likely to invest time, energy and work into delivering a FoR intervention?	SBCN’s offered the opportunity to gain psychological training to deliver a FCR intervention were largely enthusiastic and likely to invest time to train to do it.
Collective action	Will it promote or impede their work?	Projected benefits appear to be consistent with their work
	Do they think it would change the patient/SBCN relationship?	May improve interactions. Uncertainty about how patients will be approached – training will help
	Is the work compatible with the existing practices of the SBCN?	High levels of their work are focused on psychological support although low use of structured cognitive behavioural approaches
	How would the intervention impact on their workload?	The SBCNs may need to challenge their current /organisational practices in the provision of psychological support
	How does it fit with organisational goals?	
Reflexive monitoring (reflect on the trial)	How are SBCNs likely to perceive the benefits of the intervention once it has been used?	SBCN saw the benefits of intervention and understood training would be delivered. Some held concerns about the intervention being delivered via telephone and not face to face.
	Do they perceive issues associated with recruitment?	For SBCN, clear training in identification of participants with moderate FCR is required
	What would be required to make the intervention workable in practice?	There are pressures on services so choosing a regular day/time to deliver intervention will be necessary to encourage adoption into work schedule
	When would be an appropriate time to review the intervention?	

## Discussion

This is the first study to gather survey and interview data using sequential explanatory methodology to explore how specialist breast cancer nurses manage FCR, and strategies they use to identify those people at risk. It, therefore, highlights some important issues and enablers that could help embed successful implementation of this new intervention into SBCNs’ work.

FCR is increasingly reported as an unmet need within the literature [3–4, 6,-7], although the picture in clinical practice appears less clear. SBCNs are the professional group who engage frequently with people affected by breast cancer, providing a broad range of psychological support [10]. They work closely with psychological services but there appears to be no uniform approach by which they identify and stratify individuals with low, moderate or high FCR. Very few of the nurses used any formal tool, leaving much to intuition, experience and question probing**.** We found that estimates of level of FCR are at odds with self-reports from patients which suggest that need for support is not being met [14]. Indeed, this discrepancy suggests that some people may not be getting the right support at the right time, potentially compounded by a lack of systematic approaches to assessment. The Mini-AFTERc aims to bridge this need in a resource-efficient manner.

To date, a number of FCR interventions have been designed and tested [14] with most delivered to small numbers by psychologists and therapists. The care of people with breast cancer, and specifically psychological support, is not mutually exclusive to any one professional group. Although the Mini-AFTERc is a complex intervention based on a theoretical model with a detailed manual [32], and designed for SBCNs to deliver in routine practice as part of a stepped care model, it offers potential to be adapted for use by other professionals in the future. Tools to identify and manage FCR were not found to be usual practice and therefore it was significant that nurses accepted both the intervention itself and also the need to use a FCR assessment tool in their daily work.

Internationally, breast cancer guidelines recommend that people are made aware of new signs and symptoms that could signal a recurrence [33–35]. Some nurses in the interviews described discussing signs and symptoms of recurrence when asked about their confidence to discuss FCR. Their responses indicated an ease with information giving about new signs and symptoms of recurrence, rather than actively addressing psychological fears associated with the cancer recurring. When FCR is severe, it can lead to distress, difficulty coping and poorer physical health [8].

This study also gained insight into the practicality of the SBCN’s role in the delivery of the Mini-AFTERc and many were willing to consider it as a workable option that could be embedded in their practice, an important area to inform our trial design. Understandably, they reported they would require training. Listening to their training needs indicated that face-to-face sessions combined with online support was the most acceptable method. Prioritising patient care emerged as the main focus of the SBCNs work and many viewed the Mini-AFTERc as potentially beneficial to both their patients and their own professional development. Barriers to implementation included time and personal fears that introducing FCR could trigger anxiety in a person that was not previously present. Some also felt it conflicted with the positive, hopeful message about breast cancer so often portrayed [36] and that many of the nurses felt comfortable with.

The strength of this study is our mixed methods design was informed by NPT as it provided in-depth insights, before planning our current study, about how and what is required to test the efficacy of the intervention in clinical practice including confirmation that there is a high likelihood that nurses can implement the intervention as planned, they perceive a demand for this type of intervention and it fits within their practice culture and goals. This step is often overlooked in the development of trials [37], leading to assumptions being made about roles and relationships between nurses and patients that either do not exist or, as we found, are highly variable.

One limitation was our recruitment approach. We relied on a single email to a mailing list to access SBCNs. This may not have been a comprehensive list and could have included non SBCNs. Only one email was sent and we relied on the nurses both receiving and opening the email. The auto mail sifting or “junk” function that is embedded in many email programmes may have affected the number of nurses receiving the email and negatively influenced our overall responses. However, this was countered by the benefits of accessing SBCN from across the UK and in a variety of settings.

In conclusion, the Mini-AFTERc was designed specifically for patients who have completed their primary treatment for breast cancer; with minor alterations, we consider this intervention suitable for patients with other forms of cancer. By exploring views of SBCNs, a picture has emerged about how FCR conversations arise in their day-to-day work. Practices differ markedly and the self-reported skill and confidence of nurses to raise and/or respond to patients’ concerns in this area was variable. Although SBCNs recognise FCR as important and a frequent cause of distress among patients, actual techniques that assist patients manage and cope with FCR are limited. This is potentially compounded by little use of assessment tools that ask specifically about FCR. Nevertheless, SBCN expressed an interest, and willingness, to learn about the Mini-AFTERc to enhance their skills.

## Electronic supplementary material

ESM 1 (DOCX 14 kb)

ESM 2 (DOC 60 kb)

ESM 3 (DOCX 33 kb)
