# Supplementary material for: Specialist breast cancer nurses’ views on implementing a fear of cancer recurrence intervention in practice: a mixed methods study
Source: Support Care Cancer. 2019 Apr 17;28(1):201–10. doi: 10.1007/s00520-019-04762-9 (PMC6882748; doi:10.1007/s00520-019-04762-9)
Supplement: Supplementary file 1 — (DOCX 14 kb) [file 520_2019_4762_MOESM1_ESM.docx]

Online resource 1: Purposive sampling matrix

| **Participant ID** | **Age** | **Clinical area** | **How is the issue of FoR generally raised?**  1 – I always raise a discussion  10 – I only discuss FoR if the patient raises it | **How comfortable are you discussing FoR with your patients?**  1 – Extremely comfortable  10 – Not at all comfortable |
| --- | --- | --- | --- | --- |
| BCN1 | 50-59 | Surgery, Oncology | 1 | 1 |
| BCN2 | 50-59 | Oncology | 1 | 1 |
| BCN3 | 40-49 | Oncology | 1 | 1 |
| BCN4 | 40-49 | Surgery | 1 | 2 |
| BCN5 | 50-59 | Surgery | 1 | 3 |
| BCN6 | 50-59 | Surgery | 2 | 2 |
| BCN7 | 40-49 | Oncology | 2 | 2 |
| BCN8 | 50-59 | Oncology | 3 | 2 |
| BCN9 | 40-49 | Oncology | 5 | 4 |
| BCN10 | 50-59 | Surgery, Oncology | 5 | 9 |
| BCN11 | 40-49 | Oncology | 6 | 2 |
| BCN12 | 50-59 | Oncology | 6 | 7 |
| BCN13 | 30-39 | Surgery | 7 | 2 |
| BCN14 | 40-49 | Surgery | 7 | 5 |
| BCN15 | 40-49 | Surgery | 8 | 2 |
| BCN16 | 50-59 | Surgery | 8 | 3 |
| BCN17 | 50-59 | Surgery | 9 | 2 |
| BCN18 | 50-59 | Surgery, Oncology | 10 | 1 |
| BCN19 | 50-59 | Oncology | 10 | 6 |
| BCN20 | 50-59 | Primary Care | 10 | 7 |
