# Supplementary material for: Specialist breast cancer nurses’ views on implementing a fear of cancer recurrence intervention in practice: a mixed methods study
Source: Support Care Cancer. 2019 Apr 17;28(1):201–10. doi: 10.1007/s00520-019-04762-9 (PMC6882748; doi:10.1007/s00520-019-04762-9)
Supplement: Supplementary file 2 — (DOC 60 kb) [file 520_2019_4762_MOESM2_ESM.doc]

| **THEMES** | **KEY QUESTIONS** | ADDITIONAL PROMPTS |
| --- | --- | --- |
| Coherence  *Differentiation*  *Communal specification*  *Individual specification*  *Internationalization* | Could you tell me about your role as a breast care nurse and which patients you see? | What sort of care do you provide to breast cancer patients? |
|  | How is the issue of FoR generally raised? | Do you always raise the discussion, or do you only discuss FoR if the patient raises it?  How do you probe for silent concerns? |
|  | How comfortable are you discussing FoR with your patients? | What prevents you from being open about it?  What makes you comfortable? |
|  | How does this differ to your discussions around pathology and treatment options? | Do you find it easier to discuss pathology and treatment options as opposed to emotional issues and fear of recurrence? |
|  | Do you feel there is an impact on you personally when discussing FoR with patients? | Or other emotional issues with patients? |
|  | Is there any kind of support you feel would help you to better deal with FoR in your patients? | Supervision? Grief counselling? Debriefing? Continuing education? |
|  | Whose responsibility is it to discuss FoR with patients? | Is there a shared sense of purpose to address FoR among breast cancer patients? |
|  | How do you think Mini-AFTER differs to your current method of assessing patients for FoR? |  |
|  | Who do you think Mini-AFTER would benefit? | Patients? Nurses? Family members? Clinicians? |
|  | Do you think patients would value Mini-AFTER? | How would you make a clinical judgement about whether a patient values it? |
| Cognitive participation  *Initiation*  *Enrolment*  *Legitimization*  *Activation* | What sort of training would you like to receive before implementing Mini-AFTER? | Face-to-face? Online? Clinical supervision? Telephone? Shadowing an experienced practitioner? |
|  | Would you be willing to invest time to attain competence in delivering Mini-AFTER? |  |
|  | Have you recently done anything where you’ve had to obtain competence? | How did you do that? |
|  | Can you envisage Mini-AFTER changing your practice? | Are you already thinking a little more about this area? |
| Collective action – operational work  *Interactional workability*  *Relational integration*  *Skill set workability*  *Contextual integration* | Given that Mini-AFTER is designed to be incorporated into the time you already spend with a patient, how would it impact on your workload? | Would it promote or impede your work?  What impact would there be on resources such as time? |
|  | What effect do you think it would have on the support you offer women? | Would it change the patient/nurse relationship? |
|  | How do you think Mini-AFTER fits with the overall goals of your organisation? | What are their goals? Purely treatment or more holistic? |
| Reflexive monitoring – how a new intervention would affect them and those around them  *Systematization*  *Communal appraisal*  *Individual approval*  *Reconfiguration* | What may be required to make Mini-AFTER workable in practice? | Do you have access to a quiet space? |
|  | Regarding a trial of Mini-AFTER, do you perceive issues associated with recruitment? |  |
