# Supplementary material for: Specialist breast cancer nurses’ views on implementing a fear of cancer recurrence intervention in practice: a mixed methods study
Source: Support Care Cancer. 2019 Apr 17;28(1):201–10. doi: 10.1007/s00520-019-04762-9 (PMC6882748; doi:10.1007/s00520-019-04762-9)
Supplement: Supplementary file 3 — (DOCX 33 kb) [file 520_2019_4762_MOESM3_ESM.docx]

| **Coherence is the sense-making work that people do individually and collectively when they are faced with a problem of operationalising some set of practices** | |
| --- | --- |
| **Identifying FoR – how is it raised** | |
| Formal assessment | *BCN: Breast care nurses are being asked to complete things like the holistic needs assessment and tools and all kinds of things, and their biggest complaint is that it takes 45 minutes to do that. And then it just seems to get lost in the system and doesn’t seem to travel with the patient as it’s supposed to… So there’s still a lot of work to be done in that area. (17138768)* time. |
| Patient led | *I: Do you ever discuss FoR with patients then?*  *BCN: To be honest, only when it comes up. It’s patient focused rather than healthcare professional focused. (17176699)* |
| Not always addressed | *BCN: If there was a holistic needs assessment for every patient who was being discharged to open access then you could perhaps pick up more cases of fear of recurrence through that way. Unless somebody brings it up at a clinical appointment, we don’t tend to explore it. Sometimes they do and you can have a small discussion but the time we have is limited at the moment. (17183764)*  *BCN: I wouldn’t say, you’re frightened of recurrence, no. It’s usually something we talk through and they’ll say, I’m frightened of it coming back. And I wouldn’t put that in to their head. (17172978)* |
| Probing for silent concerns | *BCN: I think if I look to the holistic needs assessment when they’ve brought it in and saw that they hadn’t really ticked anything, I would say, is there anything that’s not covered that you are worried about or are concerned about or is there anything you want to ask me about. (17132110)* |
| Nurse led | *I: So thinking about fear of recurrence, is that something that you discuss with your patients at any point?*  *BCN: Yeah, we do try to at that final assessment interview, at that point that’s been an issue that’s come up. But if it hasn’t come up until that point, then we do try to discuss it, but it is a very difficult topic to help with. (17133811)* |
| Informal assessment | *BCN: Some people come to us and don't actually know what they are not coping with, so you know even if we didn't do holistic needs assessment on that person, you know, just because they've had cancer treatment in the past there would be that kind of right, let's work out what's going on here and is that an issue that you are living with from a day to day basis. (17176219)* |
| **Timing of FoR discussion** | |
| End of treatment – up to 6 months after | *BCN: It's actually when all the treatment, all the intensive treatment's over that patients must be sat at home thinking, you know, goodness me, I've been through all that and now I still don't know whether I'm cured. (17138768)* |
| Ongoing | *BCN: It’s raised throughout their patient journey really, because they’re very anxious about, at the beginning, whether the disease has moved to anywhere else in the body. And then when they come back we do a holistic needs assessment at six months where we then go through signs and symptoms to look for… And we do have ladies who phone us ten years later because they’re worried about something. (17132110)* |
| Variable | *BCN: Sometimes it is surprising the people that have struggled right the way through their chemotherapy are the ones that are super relieved it's all over and just then get on with their lives and don't think about recurrence. Whereas, you know, it's other patients who seem to cope extremely well, but then reach a bit of a brick wall after a few months, and need a lot of reassurance. (17138768)* |
| **Managing FoR (strategies)** | |
| Discussing signs and symptoms | *BCN: So, it’s trying to balance things, because most people we treat with curative intent, so we're expecting them to be cured from this, so you don't want to make people too anxious, but at the same time you want to make them aware that, well, if you get symptoms X, Y and Z, then maybe you need to get them investigated because it could be this is a sign of recurrence in the breast cancer. (18838193)*  *BCN: Or just fear of recurrence, you know, fear…oh it’s the cancer coming back. And I say, this is a normal feeling, you know, we’ll talk these symptoms through, we are listening to you and we don’t need to do these tests, but we’re listening and we talk things through. (17172978)* |
| Signposting to support group/programme | *BCN: We also run a course called Moving Forward which we do with Breast Cancer Care… So we take it in turns between the breast care nurses to run the session on breast awareness which is basically looking at signs and symptoms of possible recurrence. That often brings up quite a lot of discussion within a group or patients will come up to us afterwards and have a quiet word. (17185655)* |
| Multi-disciplinary referral | *BCN: If they need to see us, we will meet with them for psychological support and then obviously more complex patients, we’ll refer them on, if they’re out of our sphere of expertise and if they have psychiatric problems or problems that we feel…with anxiety that we can’t, you know, give them the best care, then we’ll forward them on. (17172978)* |
| Discussion of psychological aspects | *BCN: Well if they’re frightened I’ll say it’s very normal to feel like this. It’s part of, you know, what can happen. And just…so that they’re not…it’s not them…because they’ll be frightened to ring you otherwise so…’cause they feel, I don’t want to ring you. You’ll think I’m this or that. And I say, most women are afraid of it coming back and if you’re able to voices those feelings it really helps. (17172978)* |
| Open access follow-up | *BCN: We've moved to a system whereby we do a lot more open access monitoring of patients… There is a hotline number for them to call if they’re concerned about anything new… But I wouldn’t say we actively ask them, are they worried about recurrence, specifically. (17183764)* |
| Discussing prognosis | *BCN: They're given statistics as to the benefit of chemo, the benefit of the tablet medication they may have. So they're given statistics on their survival of breast cancer with and without those treatments. So they do hear that. We don't discuss it much further than that and often patients don't ask at that point. (17892573)* |
| **Confidence discussing FoR** | |
| Confident | *BCN: I don’t have any concerns about it. I think it’s more experience than anything else. The more you see patients, the more you talk to individuals, the more you find out how people are going to react. (17133811)* |
| Difficult to manage uncertainties | *I: What is it that makes those discussions about FoR so difficult then?*  *BCN: You’re taking away their hope. Because, if you’ve got secondary breast, or if you’ve got any sort of secondary cancer, your chances of it, you can be treated, but it is likely to be life limiting. And I just feel you’re taking away their hope that it won’t cause them to have an early death. (17137466)* |
| Reasonably comfortable | *BCN: Reasonably comfortable. I think, sometimes it’s a bit like the elephant in the room, it’s the thing that they want to ask, or want you to perhaps bring up. (17137466)* |
| Difficult to raise | *BCN: We don’t use the word cure but we’re doing absolutely everything to get rid of this. It’s all about reassuring. It’s all about everything’s going to be absolutely fine and to bring in the fact that, oh, actually, well, you...it might come back, you know, is actually...goes against a lot of what the message that I’d say we’re trying to get across is how I interpret it. (17495364)* |
| Unsure about how to manage fear | *BCN: I think my concern is not knowing what I could do about it. I mean, I can refer people for therapy, for counselling, but I don’t know that that’s necessarily the thing that they need. They’re not necessarily depressed or have an anxiety state, they need to know how to deal with that one particular problem. (17133811)* |
| **Responsibility to discuss FoR** | |
| All clinicians | *BCN: I think all of the clinical team because, you know, sometimes it's taking the opportunity to speak to patients. Sometimes they come to clinic for a completely different reason and that's the day that they're going to discuss their fears. I think it depends who they see, you know. (17138768)* |
| BCNs | *BCN: I’m not sure I would use the term, responsibility, but I think probably the best people would be the nurses, because I think we probably have time, I think we probably come at the patients from a bit more of an holistic approach. (17137466)* |
| **Shared sense of purpose to discuss FoR** | |
| No | *BCN: I think the surgeons don’t want to know. They just, oh it’s psychological so we’ll pass it on. And they can be quite brusque in some things and very…not all of them but there are specific ones I’m working with at the moment. It can come across as a bit harsh sometimes… The oncologists are probably better sometimes. (17172978)*  *BCN: I think there is probably a lack of confidence. The kind of self-regulation model is something that we work with, it’s our bread and butter if you like. I don’t think the clinical nurse specialists would particularly know about and have experience using this. (17176219)* |
| Unsure | *BCN: I don’t know about a shared sense of purpose, I don’t know. I think it’s more qualitative in nature isn’t it, rather than the medicalised quantitative. (17176699)* |
| **Personal impact of discussing FoR** | |
| Sometimes | *BCN: I think early on in your career, you tend to be less certain about things… So I feel as time goes on, I think you become just more competent in dealing with patients’ fears really. (17138768)* |
| Yes | *BCN: There’s always an impact personally with our type of work, you take on board peoples’ concerns sometimes even if you try hard not to. On a personal level I find experience has helped. Some if it is about talking with colleagues, peer support, some of it is just about having a life outside work and making sure that you stay healthy. (17133811)* |
| No | *BCN: No, generally not. And we are quite good in terms of we’ve got clinical supervision and a kind of outlet to be able to kind of self-care if you like. (17176219)* |
| **Support** | |
| Current support | *BCN: We have clinical supervision on a monthly basis… We have a lot of peer discussions, so if we’ve got a patient that we’re finding we need a little bit of help with or we want to make sure that we’re going in the right direction with then we’ll very openly discuss that within our team. (17132110)* |
| Missing support | *I: Do you feel like there’s any other kind of support that would help you to better deal with fear of recurrence in your patients that you’re not receiving?*  *BCN: Just some sort of tool that I could use, yeah. (17133811)* |
| **Cognitive participation is the relational work that people do to build and sustain a community of practice around a new technology or complex intervention** | |
| **Training – format** | |
| Face-to-face | *BCN: I’d probably like to watch it in action really. I think when we did the breaking bad news it was like a workshop where there was an actor where you did everything on a video. So I don’t know what would be the best way. I suppose to listen to how it works on a telephone consultation would be very interesting, and then obviously because we probably wouldn’t utilise that method, yes, to see it in a real-life situation. (17132110)*  *BCN: Well, I’ve done it in two different ways, I have done a course where a lot of it was demonstrations and I had to ask difficult questions. Another one was with an actors bit, we looked at difficult situations and how to handle those, and that was quite useful. (17133811)* |
| Online | *BCN: I'm not sure it would be more than enough for everybody, but if it was something that was online training, it would make it more accessible for people in lots of different areas. Whereas if you have a face to face training it's very much dependent on whether you can there and get the time off work, travel time and everything like this, and I don’t suppose we’re alone in being the only short-staffed, short-handed breast department. (17183764)* |
| **Training – aspects** | |
| How to manage FoR – action plan | *BCN: I think probably it would be good to know exactly what the aims of the discussions were. I think this is the other danger, isn't it, that you have these discussions, then what do you do with the information. (17138768)* |
| Advanced communication | *BCN: Well I think advance communication, difficult conversations. And I’d like to teach that sort of thing. ‘Cause I feel people that aren’t very experienced find it very, very difficult. So we do need support. Nurses do need support. (17172978)* |
| **Who to attend training** | |
| Whole BCN team | *BCN: I know from experience, at our hospital there are certain breast care nurses who have had massive amounts of advanced communication training, and others who haven't. And it's probably the ones who haven't had it that need it more, if you know what I mean. So I think it would have to be a group. (17138768)* |
| **Willing to invest time to attain competence to deliver Mini-AFTER** | |
| Yes – if adding to skillset | *BCN: Yes. I think, particularly, from the clinicians’ point of view, if they’re kind of going off for training, and if there was extra time needed to be released for them to do something like that, then yes, I think they’d want to kind of read evidence from other areas that as you say the AFTER programme. (17192301)*  *BCN: I think anything that benefits the patients, and makes the journey easier, and also gives us more skills in dealing with recurrence and fear of recurrence has to be beneficial to both the patients, the nurses, and to the service. (17137466)* |
| Available time to attend training | *I: And how much time do you get at the moment to go for training?*  *BCN: It depends. Obviously, we would have to go through our professional lead. It’s harder now than it used to be back in the day, but I think if it’s something that is going to be of value and you feel it is going to be of value professionally. I think you pick and choose now which you feel is going to be beneficial to you in your role, and your patient experience and expectation. (17175463)* |
| **Collective action is the operational work that people do to enact a set of practices, whether these represent a new technology or complex healthcare intervention** | |
| **Changing practice** | |
| Enhance practice | *BCN: Well it might enhance it, certainly. If we have a care plan or an action plan, it would probably help identify the degree of fear, probably enable us to recognise it more and put strategies in, in how we can support it. (17176699)* |
| More awareness of FoR as an issue | *BCN: I’m not a hundred per cent sure it would change it greatly, I think it would just make us more aware of what we need to address. (17132110)* |
| Fits well into current practice | *BCN: I think because we already do the holistic needs, I mean there’s no reason why we couldn't put it into like in a pack and say we offer this, but as well it's the uptake because some of our ladies don't fill in the holistic needs assessment form or don't return it to us. I guess it's the ones that do need...sometimes I'm sure we do miss some ladies that do need help but don't feel comfortable contacting us. (19948761)* |
| **Perceived difference between Mini-AFTER and current practice** | |
| More structured and specific | *BCN: I think this provides more structure because it’s around the relevant issues that, yeah, you need to address within, sort of, a holistic needs assessment. It can be very, I’d say, a bit wishy washy at times. (17175463)* |
| Highlights FoR as an issue | *BCN: I think if it’s something that you’re then broaching with people, and not necessarily because they’ve brought it up, but because it’s something that you’re asking them, it may then enable them to be able to open up about what their concerns and fears are. Which perhaps they don’t feel able to ask. (17137466)* |
| Helps to alleviate fear | *BCN: And actually, by addressing it, makes it hopefully more prevalent, and it’s dealt with at an earlier stage rather than when it becomes a real problem. (17176699)* |
| Positive impact on BCN comfort levels and support provided to patients | *BCN: I mean, what it might do, I think because we do so much of the stuff at the beginning, whether it would make us think a bit more about the whole issue of recurrence, and maybe, I don’t quite know how to put it, I was going to say, make us a bit braver. ‘Cause I think you do, but maybe you don’t always approach it. (17137466)* |
| Triaging tool | *BCN: It’s a much more structured approach obviously and it sounds like it has the potential to be able to differentiate between those patients we think we can help and those that really need a far more structured intervention or psychologist approach or therapist approach. (17133811)* |
| **Impact on support offered to patients** | |
| No impact on additional support offered to patients | *I: Do you think would it have any effect in the support that you offer women?*  *BCN: Probably not, because when we do find concerns we signpost them to wherever we feel relevant, so no, probably it wouldn’t have massive difference really, massive impact.*  *I: So the impact wouldn’t be on the support then; would the impact be more on the actual discussion itself?*  *BCN: Yeah. (17132110)* |
| Enhance support | *BCN: I think it would have an effect in terms of the level of support would be changed to what we currently do. If the level of support then means that actually you are, kind of, discharging patients off that caseload much quicker then that is good. And also, if there is, like I said, not trying to, sort of, go back to their GP and we have alleviated that side of things, it would be more beneficial. (17175463)*  *BCN: I think if something is incorporated into the strategy of follow-up for these patients that gives them early intervention and perhaps reduces the fear or makes it copeable, then actually it may have a beneficial impact on our clinics because people will be less worried about the little things that some patients will be constantly up at the breast unit for. (17183764)* |
| **Reflexive monitoring is the appraisal work that people do to assess and understand the ways that a new set of practices affect them and others around them** | |
| **Workable in practice** | |
| Time involved | *BCN: We wouldn’t have the time to do a separate session, or we wouldn’t have time to do 280 follow-up phone calls, we just don’t have the time to do that; so we would have to integrate it into something we’re already doing when we’re already seeing them. (17132110)*  *BCN: I think it would be absolutely impossible to offer it everybody because there's just too many patients, so it would have to be led by need, I think. (17138768)* |
| Timing of discussion | *BCN: I think the problem with doing it at a later stage is how you pick up that that patient is having the issues. If you think they’re alright when you do their assessment at the end of treatment and you don’t think there’s any issues, how you would pick up that they then start to have issues. And unless the patient is being particularly proactive and contacting you, then there’s going to be patients you’re going to miss. So there has to be some sort of assessment at a later stage for those ones to make sure, yes, they’re still okay. But there’s no reason why we can’t bring it up as part of the assessment interview, if they haven’t already, just as much as we talk about delayed anxiety and depression problems, we could bring it up as, this might be an issue for you, if you do find it is then you can come back to us, there are things we can try to help you with that. (17133811)* |
| Fitting in with current assessment tools | *BCN: I think we’d just adapt, I think we’d have to adapt it, we’d have to entwine them both and see how that would work really; because their thoughts and feelings are in our holistic needs assessment, we address how are they feeling about their body image and about their diagnosis; and their family, that’s addressed in relationships. So I don’t think we address expectations, so that’s something different. And the return of the cancer, that would come into their concerns I think. So I think the only one that’s probably not addressed directly is their expectation. (17132110)* |
| Delivery by staff other than BCNs | *BCN: I mean, should it be us as breast care nurses in the hospital setting because we have got a lot of experience, or should it be maybe in the community? I don't know if maybe a practice nurse would be able to do it, who has had the experience of...depending on their experience, and I certainly know in [Location] a few GP practices have actually employed an early cancer nurse. (18838193)* |
| Evidence of benefit required | *BCN: I mean we also want to treat for peoples’ cancer and we want to help them to recover, but obviously we have to think about finances and the fact that finances are limited. So, if you put initial investment into somebody then hopefully they're not going to have to access your service so much afterwards. So, that would be good, not just for us, but the person concerned. (18838193)* |
| **Format** | |
| Option of face-to-face or telephone | *BCN: if we were going to do it at the end of the surgical phase, then we could do it face to face. But if you were going to do it at a different end point, I don’t quite know how you keep up with where everybody is, but, maybe over the phone. (17137466)*  *BCN: I would like to use face, I prefer face to face interventions and I think there is lots of unspokens and, you know, and body language and just people’s unspoken behaviours that tell a story, so I prefer that but I think it would be useful to have as a phone intervention as well. (17176219)* |
| Face-to-face | *BCN: I think I would always try and go for a face to face consultation, because you can pick so many cues up, can't you, from body language, that you're going to miss out on. (17138768)* |
| **Who would Mini-AFTER benefit** | |
| Patients | *BCN: So I think there’s something missing in what we’re doing… But if there’s something that they could do in some sort of cognitive behavioural way to deal with those concerns, I’m sure that would help some people. (17133811)*  *BCN: I think you’d have to do it for everybody. But I think it would perhaps benefit those patients that have maybe been in treatment longer, and maybe would feel the effects more, of being cast adrift at the end of everything. (17137466)* |
| BCNs | *BCN: anything that we can use that helps patients helps us in the long-term. (17133811)*  *BCN: I think anything that benefits the patients, and makes the journey easier, and also gives us more skills in dealing with recurrence and fear of recurrence has to be beneficial to both the patients, the nurses, and to the service. (17137466)* |
| **Would patients value Mini-AFTER** | |
| Hopefully most would | *BCN: I guess it's not maybe for everybody. We found that with our HNAs, you know, that there is a type of person, I guess you need to want to look into that.*  *I: Yes, so of the patients that you do the HNA with do they generally value that?*  *BCN: Yes, I think so, yes it's well evaluated. (17176219)*  *BCN: Oh, I think breast cancer patients will always be happy accessing anything and everything you chuck at them. (17230248)* |
| **Patient suitability** | |
| Offer to all primary breast cancer patients | *BCN: Certainly any of our patients. I don't think there's any particular group that are more fearful than others. There's certainly a group that are more articulate than others and they're perhaps the ones you worry about, aren't they, the ones that don't express their concerns and worries. So in an ideal world, it would be great if everybody was offered this opportunity, but that would be absolutely impossible because of the number of breast patients that we see. So whether it could be something that the breast care nurses talk about and say, you know, if at any time in the future you feel as though you're feeling particularly concerned, then we can have a more in-depth conversation. (17138768)* |
| Assess which primary breast cancer patients would be suitable | *BCN: I think it would come when we’d done our six month follow up and it’s apparent from that consultation that fear of recurrence is an issue, then that’s something that you could then take on further for those patients with specific concerns… because I’ve had patients, before, that have come to me, and they’ve seen someone, and they’ve gone, oh I’m so afraid because someone said that the cancer’s going to come back, it’s coming back within two years. Well that’s something that wasn’t on their radar and it was brought to their radar and actually it was an unnecessary burden that they weren’t troubled with. (17176699)* |
| **Trial** | |
| No perceived issues conducting a trial | *BCN: I think the ideal would be to have the comparison with two to see the benefits to the Mini-AFTER.*  *I: Do you think that would be possible?*  *BCN: Yes. (17185655)* |
| No perceived issues regarding recruitment | *BCN: With all research studies, it’s explaining the project and then doing the follow-up afterwards is the easy bit, it’s recruiting them in the first place that is often the issue because they’ve got so much else going on, but I think potentially – yeah, I don’t see that there would be a big issue. Of course the network, there’s thousands of patients that potentially could go to it so you could actually reach your numbers pretty quickly. (17133811)* |
| Potential issues regarding recruitment | *BCN: I mean it would just be whether or not we could get that patient group, so obviously we would need to be referred to, whereas, if it was a breast care nurse and that’s their patient group there. (17176219)* |
| Cooperation of organisations | *BCN: I think they’d want to kind of read evidence from other areas that as you say the AFTER programme. Something like kind of hard statistics, hard evidence. (17192301)* |
| Time involved | *BCN: I can see benefits to doing it. It would just be like numbers, how realistic is the 30 minutes because I know we get ladies coming in saying, I just want a 30-minute appointment, and an hour later they’re still with us and that’s important to them but if a half an hour becomes an hour it’s then tricky. I think afterwards as well in terms of what that then means in terms of half an hour with a patient, how much is that then writing up and signposting to others, you know, it’s not just half an hour, is it? (17185655)* |
